# Learning analytics in higher education: a preponderance of analytics but very little learning?

**DOI:** 10.1186/s41239-021-00258-x

**Published:** 2021-05-04

**Authors:** Carolina Guzmán-Valenzuela, Carolina Gómez-González, Andrés Rojas-Murphy Tagle, Alejandro Lorca-Vyhmeister

**Affiliations:** 1 Facultad de Educación y Humanidades, Universidad de Tarapacá, Arica, Chile; 2 Universidad de Chile, Santiago, Chile; 3 Universidad de Valparaíso, Valparaíso, Chile

**Keywords:** Learning analytics, Higher education, Challenges, Learning

## Abstract

In a context where learning mediated by technology has gained prominence in higher education, learning analytics has become a powerful tool to collect and analyse data with the aim of improving students’ learning. However, learning analytics is part of a young community and its developments deserve further exploration. Some critical stances claim that learning analytics tends to underplay the complexity of teaching-learning processes. By means of both a bibliometric and a content analysis, this paper examines the publication patterns on learning analytics in higher education and their main challenges. 385 papers that were published in WoScc and SciELO indexes between 2013 and 2019 were identified and analysed. Learning analytics is a vibrant and fast-developing community. However, it continues to face multiple and complex challenges, especially regarding students’ learning and their implications. The paper concludes by distinguishing between a practice-based and management-oriented community of learning analytics and an academic-oriented community. Within both communities, though, it seems that the focus is more on analytics than on learning.

**Supplementary Information:**

The online version contains supplementary material available at 10.1186/s41239-021-00258-x.

## Introduction

For the last twenty years or so, the introduction of learning analytics (LA) in higher education has prompted new research approaches to teaching and learning (Viberget al. 2018). Although LA is being used by numerous higher education institutions, it has been considered an emergent field of study and deserves more exploration (Daniel 2015; Leitneret al. 2017; Peña-Ayala 2017; Wong 2019). Further, it has been argued that LA has a reduced understanding of education and so underplays the complexity of teaching and learning processes (Lundie 2017; Selwyn 2015, 2019).

In 2012, Ferguson published a seminal paper about the developments and challenges of LA broadly between their origins from around 2000 and 2010. By analysing the references contained in the 70 papers submitted to the first conference on LA held in 2012, her paper addressed LA’s drivers, differentiation with other analytic communities and challenges. One of Ferguson’s arguments was that in:

Tracing the development of learning analytics… [it is possible to] highlight a gradual shift away from a technological focus towards an educational focus; the introduction of tools, initiatives and methods that are significant in the field today; and the issues that have not yet been addressed (Ferguson 2012, p. 305).

Eight years later, however, some dimensions of LA as a field of study remain underexplored and new challenges seem to emerge, particularly at the higher education level,  including issues about the research methodologies used to analyse and interpret data—which have been predominately quantitative oriented (Leitner et al. 2017; Viberg et al. 2018). Further, new and tighter regulations around privacy and confidentiality in higher education institutions restrict the collection of data (Khalil et al. 2018). Additionally, the extent to which LA investigates learning and use educational theories to interpret data analytics remains unclear (Selwin 2019; Wong 2019).

By means of both a bibliometric and a thematic analysis of papers on LA *within* the field of education published between 2003 and 2019 and inspired by the study conducted by Ferguson (2012), this paper addresses the main challenges and limitations around Leaning Analytics in higher education with a focus on learning processes. The research questions guiding this study were: (i) what are the main publication patterns of papers on LA and higher education contained in Web of Science (WoScc) and SciELO journals? (ii) Which are the main methodologies in use in conducting research on LA in higher education? (iii) Do these publications have an educational focus as stated by Ferguson (2012)? and (iv) Are there challenges and concerns regarding LA not so far identified in the literature?

The contribution of this paper to the understanding of LA and its connection with learning and educational theories is unique in being focused on papers indexed in the research categories connected with the field of education (and specifically in higher education) as used in both the WoScc and SciELO indexes^1^. A quick search of papers about LA published between 2013 and 2019 shows that the overwhelming majority of papers about LA published in both WoScc and SciELO indexes are not connected with the field of education (more than 65, 000 papers).

### Learning analytics: key definitions and developments

Generally speaking, LA is a data-driven approach in managing systems which allows the gathering of large amounts of data generated by students in order to predict their individual learning outcomes (Fynn 2016; Peña-Ayala 2017). LA aims at generating metrics and quantifiers of students’ behaviours and gaining awareness about the types of interventions that might be helpful in improving student learning (Jones 2019). The classic work by Campbell et al. (2007) sets out five steps for LA: Capture, Report, Predict, Act, Refine. The cycle starts with learners, who generate data, which is processed into metrics, which are used to inform interventions, which in turn, affect learners.

Since 2011 onwards (Waheed et al. 2018), LA has gained traction with an eruption of on-line learning and learning management systems (LMS)—also known as virtual learning environments (VLE)—which have made available a considerable volume of data about learning (Leitner et al. 2017). This way, ‘*Every page visited, every interaction, every click *[*by students*]* can in theory be recorded and stored*’ (Clow 2013, p. 685). In turn, these data can be collected, digitalised, and analysed through complex statistical and computational tools. As a result, many higher education institutions have adopted LA to collect and analyse student data, which, in turn, has facilitated a series of mechanisms to improve learning not only at an institutional level, but also at national levels (Peña-Ayala 2017).

Currently, and especially because of the Covid-19 pandemic, many courses in higher education institutions offer either on-line courses or a blended approach to learning that combine lectures with self-regulated learning activities organised in an institutional learning management system (LMS) such as *Blackboard* and *Moodle*. These LMS offer a fertile ground for LA since data can readily be mined from them (Conde et al. 2018; Peña-Ayala et al. 2017). Another data source for LA are on-line learning systems such as massive open online courses (MOOCs) (Khalil et al. 2018). Such an analysis can also be complemented with socio-demographic information, course engagement data from students (Scholes 2016), grades of entrance and examination tests, and library usage (Ifenthaler and Schumacher 2016).

In order to process large amounts of student data, LA relies on mathematical and computational tools through techniques such as classification, clustering, text mining, and visualisation (Ifenthaler and Gibson 2020; Peña-Ayala 2017). Statistical techniques include decision trees, neural networks, and Bayesian networks (Ifenthaler and Gibson 2020; Peña-Ayala et al 2017). These techniques are frequently complemented with regressions, correlations, and other analyses (Sergis and Sampson 2017).

LA incorporates a particular timescale in analysing the learning process. Whereas educational research has been traditionally concerned with long periods of student development (typically semesters or years), LA techniques are capable of capturing learning processes moment-by-moment (Molenaar et al. 2019), for example, students completing on-line tasks, working in groups, or interacting with multiple university systems (such as libraries and academic tutoring units). This information can be obtained and processed in real time, facilitating immediate decision making (Ifenthaler and Yau 2020).

Until very recently, the LA community has been largely a practice-based community led by institutional researchers and managers with interests in data visualisation, instructor feedback, student recommendations, student performance predictions, student mental models and detection of unwanted behaviours (Daniel 2015; Peña-Ayala et al. 2017; Wong 2019). The ultimate goal has been preventing non-completion in higher education institutions (Şahin and Yurdugül 2020) although it is also hoped that LA can help students better to reflect on and plan their learning activities (Peña-Ayala et al 2017).

Significantly, the practical community of LA also includes intervention strategies for students identified as at risk (Daniel 2015; Fynn 2016; Wong 2019). In recent years, its application has extended to other areas including support for active methodologies based on problems (Saqr and Alamro 2019), decision making and interventions in the classroom (Molenaar et al. 2019), at an institutional level in relation to meta-data (Jia and Maloney 2015), or the understanding of self-regulated learning (Blackmon and Moore 2020; Wong 2019).

### Learning analytics: some historical notes

In tracing back the developments of LA, the literature shows that educational data mining (EDM) has had an important role especially in North America, Western Europe and Australia/New Zealand (Ferguson 2012). EDM emerged in the early 2000s from the analysis of logs produced by student interaction with learning management systems or on-line programmes through mathematical and computational tools (Romero and Ventura 2013).

EDM has been used to enhance web-based learning environments for the educator to better evaluate students’ learning processes and prioritise and design educational interventions as well as for the learners to help them in their learning endeavours (Aldowah et al. 2019; Daniel 2015). In its beginnings, though, EDM had a data-driven approach to learning that, according to Ferguson (2012), inspired by social-constructivism theories proposed by Dewey and Vygotsky, rapidly moved toward a more educational-driven approach focused on understanding and optimising learning.

Although EDM and LA share many characteristics, there are differences between these two research programmes (Aldowah et al. 2019). While EDM privileges the automated discovery of patterns based on individual components and the interaction between them (Peña-Ayala et al. 2017), LA focuses on expert judgment and tests hypotheses with the help of automatic discovery models (Ifenthaler and Gibson 2020; Peña-Ayala 2017). Consequently, the models generated by EDM are usually used to develop intelligent tutoring systems, while those of LA tend to support processes of decision making by administrators and institutional researchers.

LA and EDM communities overlap and boundaries among them are fuzzy. In this paper, Learning Analytics is conceived as related to teaching and learning issues and the way in which students’ learning in higher education might be improved.

### Challenges for learning analytics

Some researchers have criticised LA and on various grounds. One concern is that LA has been developed without the active participation of students and teachers (Ferguson 2012; Mor et. al. 2015; Sergis and Sampson 2017; Selwyn 2019). Usually, central  institutional units are in control of the processes of gathering and analysing the data with students and teachers being relegated to an observational role (Leitner et al. 2017; Tsai and Gasevic 2017) and having scant understandings of the LA techniques being put into use (Selwyn 2019).

A second major area of concern in the literature relates to the students and their learning. Students, it appears, are rarely consulted in the development of LA systems in universities (Ferguson 2012; Lundie 2017). Moreover, it is a key principle of LA techniques that they identify students at risk and there are concerns in the literature that so identifying students might promote their labelling (Scholes 2016; Wintrup 2017) and act as self-fulfilling prophecies, so exacerbating on the very problems that LA is designed to reduce. Academic staff might also be trapped in these processes, coming to hold unduly limited expectations about students’ academic success.

A third critique has noted that institutions’ interests in grades, persistence and non-completion metrics tend to prevail over students’ motivation, engagement and satisfaction and more formative assessments of learning (Lundie 2017). It is not clear, therefore, that LA are having a positive effect on learning (Gašević et al. 2016; Scheffel et al. 2014; Rogers et al. 2016; Viberg et al. 2018). In this respect and by means of a systematic literature review on LA in higher education, Viberg et al. (2018) found that only a small proportion of research papers (9%) show evidence that LA improve higher education students’ learning outcomes understood as knowledge acquisition, skill development or cognitive gains. Furthermore, in conducting a literature review, Leitner et al. (2017) found that most of the papers were about researchers and administrators making decisions accordingly rather than teachers and students.

There are also research concerns about LA and the methods to analyse and interpret data. Some years ago, Clow (2013) observed that LA adopts a rather eclectic approach to learning and usually rely unreflectively on techniques and methods that are not articulated and lack an explicit theory. Also, according to some authors, LA underplays the complexity of teaching-learning processes (Lundie 2017; Selwyn 2015, 2019; Wilson et al. 2017). As Subotzky & Prinsloo observed:

Only a relatively low proportion of student success variation can be explained by traditional statistical modelling techniques such as multiple linear regression analyses. These techniques simply establish valid and reliable relationships between relatively few variables relevant to a specific context (Subotzky and Prinsloo 2011, p.183).

LA collects and measures what is readily available, can be measured and analysed most easily (Selwyn 2015, 2019) and, if students are disinclined to engage in processes of active learning, the data that are collected and analysed are easily misinterpreted (Mor et. al. 2015). Further, Selwyn (2015, 2019) points to an improper use of LA. He observes LA data have a ‘social life’ with its ‘diverse sets of raw data… being continually combined and recombined, with different data entities produced from varying iterations and calculations’ (Selwyn 2015, p.70). Instead, here, the way opens to the development of protocols to regulate how data can be used within educational institutions across specific contexts and timeframes (Daniel 2015; Rogers et al. 2016).

The use of LA has also raised concerns about privacy and vulnerability (Daniel 2015; Jones 2019; Viberg et al. 2018). LA might be seen as a tool of surveillance through which students are permanently observed (Wintrup 2017). Complementarily, this can be understood as a limitation of students’ freedoms (Wintrup 2017). Through machine-driven algorithms, governments and institutions might steer students’ choices, even across their lifeworld (Fynn 2016; Lundie 2017). Also, questions arise about who collects the data, where they are stored, who is accountable for them, the extent to which they are secured, and what is going to be done with them (Ifenthaler and Schumacher 2016; Slade and Prinsloo 2013).

## Methodology

### Sample selection

A search of papers about learning analytics in higher education in two databases—WoS Core Collection Index (WoScc) and WoS SciELO Citation Index (SciELO)—and published between 2013 and 2019 was conducted on August 9th and October 10th, 2020 and revised on December 28th, 2020.

As mentioned, this paper was inspired by Ferguson’s (2012) study about the main challenges and limitations of LA. However, and considering that that paper considered only papers submitted to the first conference on LA in 2012, it was decided to widen the scope of this analysis to include papers contained in journals in two well-known indexes: WoScc and SciELO.

While WoScc is known for containing the most prestigious journals across disciplines (Vessuri et al. 2014), SciELO is a popular index in Latin America and South Africa (Alperin et al. 2011). In including SciELO papers, it was intended to increase representation of papers published in peripheral regions such as Latin America and Africa. Further, both WoS and ScieLO indexes are searchable under the Clarivate Analytics’ Web of Science website which facilitates the search and allows the use of the same categories and terms. While WoScc requires journals to be indexed in English only (regardless of the language the paper is written in), SciELO can be indexed in different languages, mainly English, Portuguese and Spanish. Given the main aim of this paper, only papers contained in journals within the field of education were considered for analysis.

The journal data were filtered in the following way: Year: 2013 to 2019. For WoS, flagship core collection^2^: Scientific Citation Indexing Expanded (SCIE), Social Sciences Citation Index (SCI) and Arts & Humanities Citation Index (A&HCI). For both WoScc and SciELO: Selected Research Categories: Education & Educational Research, Education Scientific Disciplines, Psychology Educational and Education Special.

Following the literature review, the search terms used to identify eligible publications were: ‘learning analytics’, ‘big data’, ‘data mining’ and ‘machine learning’. In order to accommodate indexing protocols in SciELO, the search included keywords in English, Spanish and Portuguese (In Portuguese: ‘análise pedagógica’, ‘análise de aprendizagem’, ‘aprendizado de máquina’, ‘aprendizagem de máquina’, ‘megadados’, ‘grandes dados’, ‘mineração de dados’ and ‘extração de dados. In Spanish: ‘Analítica de Aprendizaje’, ‘datos masivos’, ‘aprendizaje automático’ and ‘Minería de datos’).

Only papers—either theoretical or empirical, including systematic reviews—were considered in the sample so that proceedings, chapters in books or other type of document were discarded. Titles and keywords were screened several times by three researchers individually and only papers about higher education (including MOOCs) were considered for analysis. In case of doubt, the abstract was read; if the doubt persisted, the whole paper was read by the researchers individually to discard papers focused on other educational levels. This process ensured the reliability of the sample. As a result, 375 papers for WoScc and 10 for SciELO were identified (385 papers in total).

### Data analysis

Two different analyses were conducted, a bibliometric analysis and a thematic analysis. Bibliometric analyses provide descriptive statistics regarding the most significant publication trends on a specific topic (Bornmann and Mutz 2015). In this study, a bibliometric analysis of the selected papers (385) was performed so as to identify the evolution of number of publications, affiliation of the authors, journals of publication and methodologies used (for empirical papers).

Thematic analysis is a type of qualitative analysis that aims to establish recurrent and relevant themes (Ayres 2008). Such an analysis of 20 papers, addressing critical issues and challenges of LA in higher education (5% of the total papers), was carried out by the team (see Fig. 2). In order to conduct this analysis, a deductive-inductive process was followed. The deductive categories were defined in the light of the literature review and included the following themes: educational theories, stakeholders, ethical issues, methods and data analysis. Additionally, inductive categories including data governance, structural factors and research results emerged from the analysis (for detailed definition of each category, see Table 4).

### Outcomes

#### Number of publications on LA in higher education by year, country of affiliation of first authors, journal, and language of publication for WoScc and SciELO databases

**Fig. 1:**
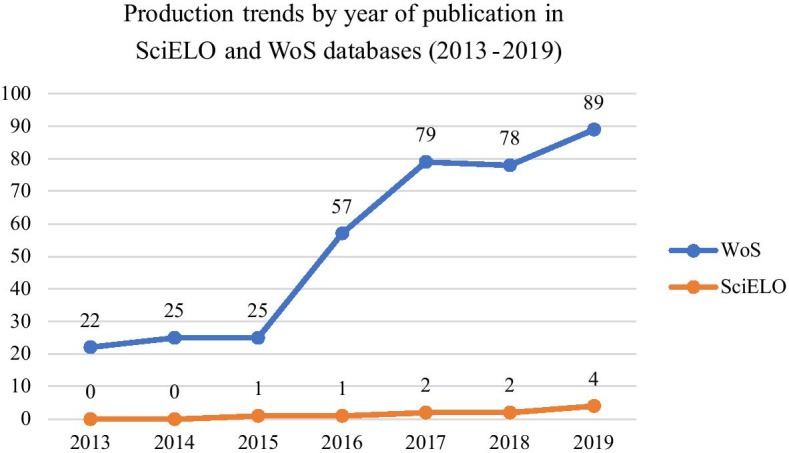
Number of publications per year in WoScc and SciELO databases between 2013-2019. Own source

Fig. 1 shows the number of papers published between 2013 and 2019 for WoScc and SciELO. The number of publications is much higher in WoScc than SciELO. However, in both databases, there is an upward trend in publications on LA. In 2013, WoScc included 22 papers and reached 89 publications in 2019. In SciELO, the number of publications, although negligible, shows an increase across the years, reaching 4 papers in 2019. For both indexes, therefore, there is a four-fold increase.

Regarding the first author affiliations (Table 1), there are 48 countries represented in the sample, with the USA being the leading country. Next are Spain, UK, Australia, China and Taiwan. The remaining countries have marginal publication percentages. Regarding the language of publication, 97.1% of papers were written in English, 2.6% in Spanish and one paper in German (0.3%).

**Table 1:** Publications per country of affiliation of the first author in SciELO and WoScc databases (2013–2019)

Country	Number of publications	%
USA	78	20.3
Spain	45	11.7
UK	37	9.6
Australia	31	8.1
China	28	7.3
Taiwan	22	5.7
Canada	12	3.1
Netherlands	11	2.9
Serbia, South Korea^*^	9	2.3
Mexico	8	2.1
Germany	7	1.8
*Japan, Norway, South Africa*^a^	6	1.6
*Israel, Turkey*^a^	5	1.3
*Greece, India, New Zealand*^a^	4	1.0
*Belgium, Finland, Ireland, Pakistan, Saudi Arabia, Tunisia*^a^	3	0.8
*Austria, Chile, Colombia, Cyprus, Portugal, Romania, Sweden, united Arab Emirates*^a^	2	0.5
*Algeria, Bosnia and Herceg, Brazil, Costa Rica, Ecuador, Fiji, Iran, Italy, Lithuania, Malaysia, Montenegro, Peru, Switzerland, Thailand*^a^	1	0.3
*Total*	385	100

^a^These countries have been grouped by number of publications, each having the same number expressed in the number of publications column, and its corresponding percentage in the next column

Own source

The selected papers were published across 84 different WoScc and 9 SciELO journals. Table 2 shows the journal of publication classified according to the scope of the journals. Both the aims and scope of each journal were read and then grouped into the following five clusters: Education & Technology; Education; Education & Engineering; Medical Education; and Other. Papers published in Education & Technology prevail.

**Table 2:** Publications per Focus of the Journals in SciELO and WoScc databases (2013–2019)

Focus of the journal	Journals	Number of publications	%
Education and Technology	Computers & Education; British Journal of Educational Technology; Educational Technology & Society; Interactive Learning Environments; Internet and Higher Education; Etr&D-Educational Technology Research and Development; Journal of Computer Assisted Learning; Australasian Journal of Educational Technology; Journal of Computing In Higher Education; Acm Transactions on Computing Education; Computer Assisted Language Learning; Journal of Educational Computing Research; International Journal of Computer-Supported Collaborative Learning; Learning Media and Technology; International Review of Research in Open and Distance Learning; International Journal of Technology And Design Education; Journal Of Science Education and Technology; System; Recall	174	45
Education	International Review of Research in Open And Distributed Learning; International; Journal of Educational Technology in Higher Education; Eurasia Journal of Mathematics Science and Technology Education; Educational Sciences-Theory & Practice; Distance Education; Physical Review Physics Education Research; Higher Education; Assessment & Evaluation in Higher Education; Teaching in Higher Education; Croatian Journal of Education-Hrvatski Casopis Za Odgoj I Obrazovanje; Research In Higher Education; Active Learning in Higher Education; Cadmo; Journal of Higher Education; Studies in Higher Education; Applied Measurement In Education; Studies In Educational Evaluation; Higher Education Policy; Journal of Educational Measurement; Thinking Skills and Creativity; British Educational Research Journal; Asia Pacific Education Review; Learning And Individual Differences; Cultura y Educacion; Journal of the Learning Sciences; Comunicar; Teachers College Record; Zeitschrift Fur Erziehungswissenschaft; Review of Higher Education; Innovations in Education and Teaching International; Ride; Revista Iberoamericana para la Investigación y el Desarrollo Educativo; Revista Electrónica de Investigación Educativa; Educación y Educadores; Estudios Pedagógicos; Innovación Educativa; Diálogos Sobre Educación. Temas Actuales en Investigación Educativa; Conrado; Revista Electrónica Educare; Apertura	88	22,7
Education and Engineering	Ieee Transactions on Learning Technologies; Computer Applications in Engineering Education; International Journal of Engineering Education; Ieee Transactions on Education; International Journal of Electrical Engineering Education.	87	22,5
Medical Education	Bmc Medical Education Medical Teacher; Academic Medicine; Advances in Physiology Education; Anatomical Sciences Education; European Journal of Dental Education; Teaching and Learning in Medicine; American Journal of Pharmaceutical Education; Journal of Continuing Education in the Health Professions; Medical Education; Journal of Surgical Education	23	5,9
Other	Journal of Chemical Education; Journal of Educational Psychology; Cbe-Life Sciences Education; Journal of Geography in Higher Education; Biochemistry and Molecular; Biology Education; Assessing Writing; Language Learning; Physical Review Special Topics-Physics Education Research; Language Teaching; Educational Psychology; Journal of Hospitality Leisure Sport & Tourism Education	13	3,4
Total		385	100

Own source

### Methodologies

Table 3 shows the methodologies used in the identified empirical papers. It is observed that quantitative methodologies prevail.

**Table 3:** Papers published per methodology used in SciELO and WoScc databases (2013−2019)

Methodology	SciELO		WoScc	
	Number of publications	%	Number of publications	%
Quantitative	6	60.0	293	78.1
Qualitative	0	0.0	18	4.8
Mixed methods	0	0.0	27	7.2
Non-empirical	4	40.0	37	9.9
Total:	10	100.0	375	100

Own source

### Thematic analysis

**Fig. 2:**
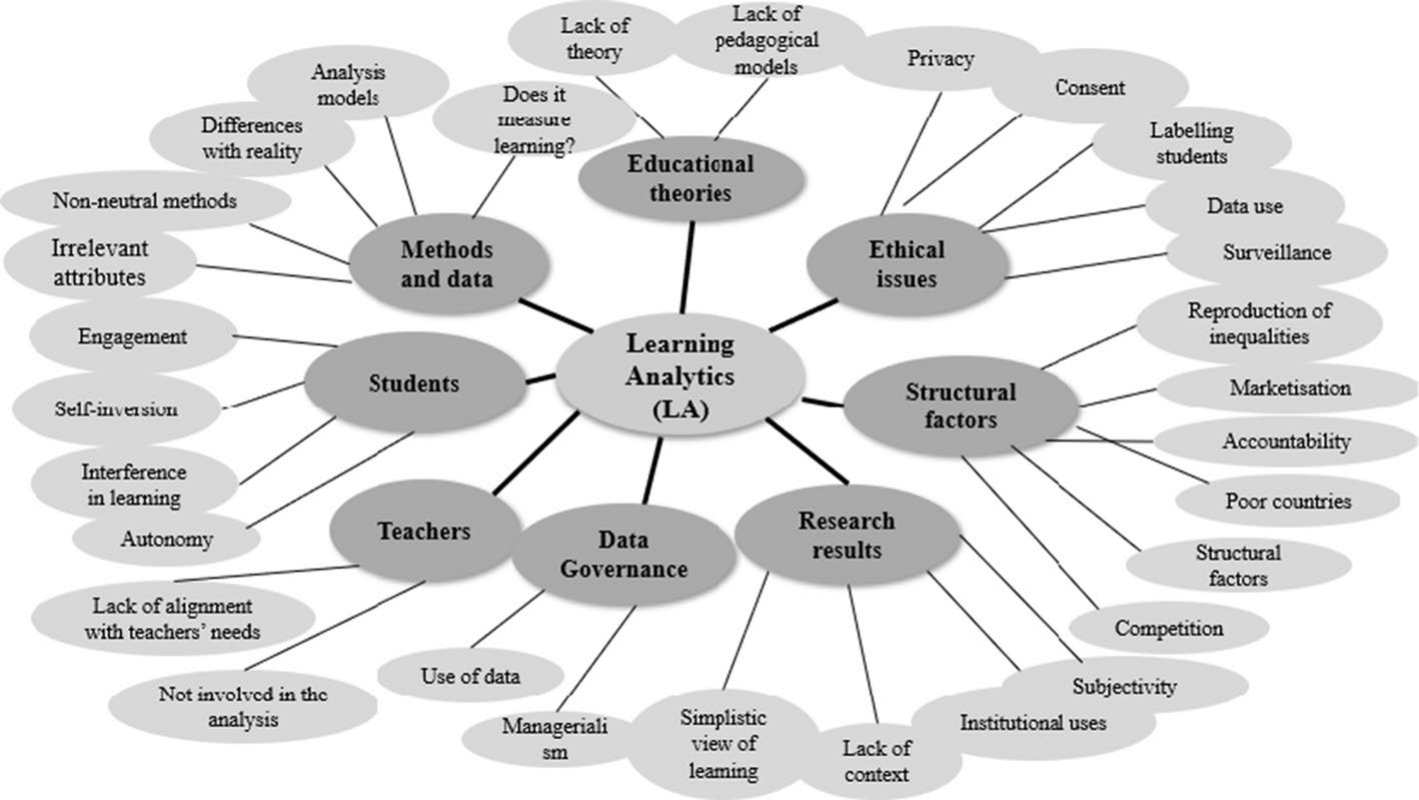
Critical issues and challenges for learning analytics. Own source

In the papers tackling critical issues in LA and higher education, numerous concerns have been voiced, some of which were identified in the earlier literature review here, while others are new, emerging through this thematic analysis. Some of these concerns relate to teaching-learning processes while others relate to research in LA (including theories and methods as well as results and their impact). Other key issues were: ethical and privacy issues involved in the data collection; the impact of LA; the link between data and policies (at different levels); and more structural factors (social, financial and political). Fig. 2 shows the codes (in outer circle) grouped into categories of analysis (in dark grey).

Table 4 lists the eight subordinate categories of analysis drawn from the targeted papers and, against each one, offers a description. In each case, the description indicates the codes that constitute the category in question. The third column lists the papers from which the codes and categories were drawn. The most salient categories are discussed in the section which immediately follows.

**Table 4:** Critical issues and challenges for learning analytics

Category	Description	Papers
1. Students	Learning design and the extent to which technologies may interfere in students’ learning (their autonomy, engagement, the ways in which they invest their time and effort and their progress in their learning)	(Bodily and Verbert 2017; Clow 2013; Jones and McCoy 2019 Perrotta and Williamson 2018; Selwyn 2015; Wintrup 2017)
2. Teachers	Lack of alignment between teachers’ pedagogical activities and LA. Also, the detachment between teachers and those responsible for LA (managers and administrators)	(Rojas-Castro 2017; Scheffel et al. 2014; Selwyn 2015;)
3. Educational theories	Lack of educational and pedagogical theories underpinning LA	(Avello and Duart 2016 ; Clow 2013; Perrotta and Williamson 2018; Rambe and Moeti 2017; Schwendimann et al. 2016)
4. Use of methods and data analysis	Use of highly technical mathematical models and quantitative techniques that include irrelevant attributes. Also, that the management of such large data sets is unduly time-consuming. Also, concerns about the ‘neutrality’ of data collection and techniques of analysis and the ways in which certain methods produce data which might affect results and have an impact on students’ learning. Finally, a concern about whether the methods actually measure learning	(Bodily and Verbert 2017; Clow 2013; Dawson and Siemens 2014; Johanes and Thille 2019; Jones and McCoy 2019; Perrotta and Williamson 2018; Prinsloo 2019; Selwyn 2015; Urbina and De la Calleja 2017; Williamson 2019; Wintrup 2017)
5. Research results	Diverse concerns about the results produced by LA. For example, the reduction of the complexities of learning into data; the lack of consideration of other learning factors or the broader context that cannot be measured; the loss of subjectivity and other factors involved in learning processes; the non-regulated cross-border use of data; and the ‘ecological validity’ of data	(Dawson and Siemens 2014; Jones and McCoy 2019 ; Khalil et. al. 2018; Perrotta and Williamson 2018; Selwyn 2015; Timmis et al. 2016; Watson et al. 2017; Williamson 2019)
6. Data governance	Ways in which data are managed and used at micro (classroom), institutional and macro (national policies) levels so as to improve teaching and learning. Also, the lack of understanding about what to do with or how to use data. Also, a ‘managerialist’ approach to LA.	(Johanes and Thille 2019; Perrotta and Williamson 2018; Selwyn 2015; Williamson 2019; Wintrup 2017)
7. Ethical issues	Issues of privacy, confidentiality, informed consent, surveillance, and labelling students at risk	(Bodily and Verbert 2017; Johanes and Thille 2019; Khalil et al. 2018; Pardo and Siemens 2014; Scheffel et al. 2014; Selwyn 2015; Timmis et al. 2016; Williamson 2019; Wintrup 2017)
8. Structural factors	Structural concerns: commercial use of data or business-like practices; material conditions (technology) in using LA, especially considering countries with less-developed economies; a heightening of accountability processes; increasing competition among institutions; promotion of social inequalities and other exclusionary practices (for example, MOOCs promoted by Western universities in poor countries). Also, financial, political, philosophical, epistemological and technical-mathematical aspects being characteristically absent	(Johanes and Thille 2019; Perrotta and Williamson 2018; Rambe and Moeti. 2017; Selwyn 2015; Williamson 2019)

Own source

## Discussion

We take the research questions in turn:

### (i) What are the main publication patterns of papers on LA and higher education contained in Web of Science (WoScc) and SciELO journals?

LA has generated a vibrant research programme in higher education. The increase in the number of publications in education and educational research between 2013 and 2019 indicates a heightened research interest in the matter. However, most of these publications are contained in WoScc journals, in English, with lead authors from countries in the global North (mainly Anglo-Saxon and European countries). This is not surprising considering that most of the WoScc journals are published in English (Chavarro et al. 2017). Also, this result is aligned with the literature that indicates that countries like the United States, Spain, Australia, and the UK are the top countries in investigating and publishing papers on LA (Waheed et al. 2018).

Furthermore, these papers tend to appear in the more prestigious journals (those of the WoScc). There are three possible explanations for this trend: *First*, that countries with more developed economies have better technological infrastructures that facilitates the promotion of more virtual and blended learning within their universities which, in turn, generates possibilities for data management systems and, thence research. *Second*, in countries with less-developed economies, there are projects to develop virtual learning environments and LA initiatives, but their impact and results have yet to be investigated and published in scientific papers. *Third*, publications directed at LA are appearing in countries with less-developed economies, but they appear in journals with less visibility than the WoScc index offers. Also, those publications tend not to be published in English so adding to their invisibility (Guzmán-Valenzuela and Gómez 2019). A combination of all these possible explanations is likely.

Although all the selected papers in this study fall within the field of education and educational research, most of the papers (more than 50%) were published in journals attached to engineering and technology (both being applied sciences). In other words, journals in the broader fields of education tend to publish fewer papers on LA in higher education. This might be related to the third research question discussed below.

### (ii) Which are the main methodologies in use in conducting research on LA in higher education?

Most of the empirical studies identified in this study are quantitative in nature (78.1% WoScc papers and 60% SciELO papers). A trend towards the use of quantitative methodology has been largely acknowledged in the literature (Ifenthaler and Gibson 2020; Peña-Ayala et al 2017; Viberg et al. 2018). LA research commonly deploys an approach focused on the data themselves or on mathematical models by which to process them (Peña-Ayala 2017). A response to this limitation has been given by Selwyn (2015, 2019) who has pointed that what is collected and measured by LA is what it can be realistically analysed.

### (iii) Do these publications have an educational focus as stated by Ferguson (2012)?

This question cannot be addressed in absolute terms since the sample here considered is limited. However, from the thematic analysis (Fig. 2 and Table 4), questions and concerns about LA and its educational focus as stated by Selwyn (2015, 2019) remain. This is especially so regarding teaching and learning processes and the extent to which the data collected around the students’ interactions with a learning platform (for example, the number of documents downloaded, of participations in forums, of times the students have accessed the platform in a span of time, and so on) correspond to actual learning on the part of the student and ways of improving it.

The key concerns for LA in higher education, identified in the thematic analysis, are related to the extent to which:

- Learning design and the use of LMS facilitate students’ learning (autonomy, learning progress, time investment, effort, engagement) (Clow 2013; Bodily and Verbert 2017; Jones and McCoy 2019; Perrotta and Williamson 2018; Selwyn 2015, 2019; Wintrup 2017). A way to actively involve students in the design of learning environments might consist of asking about their perceptions of the LMS, their engagement with the tasks and their perceptions about their learning processes (Ferguson 2012; Lundie 2017).
- Teachers are involved in the design of the learning environments and the extent to which there is an alignment between the teaching and learning activities and the assessment tasks that count for LA (Rojas-Castro 2017; Selwyn 2015, 2019; Scheffel et al. 2014).
- Specific learning contexts (national, institutional, disciplinary contexts) where learning takes place are considered for analysis (Daniel 2015; Selwyn 2015, 2019; Timmis et al. 2016).
- LA oversimplifies the learning process by making it equivalent to observable behaviours (for example, the number of times that students download documents or access the LMS) (Dawson and Siemens 2014; Jones and McCoy 2019; Khalil et al. 2018; Perrotta and Williamson 2018; Selwyn 2015, 2019; Timmis et al. 2016; Viberg et al. 2018; Watson et al. 2017; Wilson et al. 2017).
- LA is focused on collecting and analysing large sets of data in the light of educational and pedagogical theories (Avello and Duart 2016; Clow 2013; Perrotta and Williamson 2018; Rambe and Moeti. 2017; Schwendimann et al. 2016; Selwyn 2015, 2019). The LA literature rarely identifies instances where educational or pedagogical paradigms are drawn upon in illuminating students’ learning processes (Selwyn 2015, 2019).

### (iv) Are there challenges and concerns regarding LA in the literature not so far identified?

While some challenges and concerns can be considered as long-standing, others are new and warrant further examination. Besides the issue of learning, there is a host of issues and concerns in front of the developments of LA in higher education. In this respect, and in the light of the thematic analysis, some issues that deserve more exploration are:

- Guaranteeing that the methods and statistical techniques associated with LA are neutral-free and do not secrete a bias in relation to students’ learning.
- Acknowledging that—if students are aware that they are being observed and their behaviours are being measured-the virtual learning environments may prompt students to develop strategic behaviours and distort generated data.
- Accepting that  data generated and collected in specific educational settings are not context-free and cannot be generalised and straightforwardly applied to other contexts, in different moments.

Furthermore, critical literatures on LA raise concerns at all levels of educational systems, from the individual learner through institutions to the national policy framework. It follows that there are also implicit issues as to ways in which the different stakeholders—students, teachers, developers, managers, and authorities-can be brought more into collaborative discussions so as to mitigate concerns over LA. For example, that students are being measured and monitored without their consent or without respecting their privacy; that teachers’ approaches to their own teaching are not being taken into account; that data are being used for purposes other than learning; that the focus of LA is on mathematical models rather than educational aims; and that authorities have not fully worked through the data that they are amassing.

Part of these problems might be a consequence of two LA communities—a data driven, practical and management-oriented community focused on interventions, and an academic community more focused on theories and their development—that tend not to work together (Clow 2013; Khalil et al. 2018; Selwyn 2015, 2019; Wong 2019).

Finally, there are some structural dimensions that deserve consideration when conducting research on learning analytics since such dimensions may have an unintended impact:

- LA and its use for commercial aims, comparisons, and audit cultures: these challenges remind us that data can be manipulated and used with unintended consequences.
- LA might reinforce learning inequalities and the North/South imbalance. In this perspective, LA might be seen as exercising  power and be  a hegemonic tool that reinforces inequalities between wealthy and poor countries.

## Conclusion

The study presented here shows an upward trend in the number of papers on LA in higher education. The results also show that most of these papers are published in journals attached to Engineering and Technology, that they tend to use quantitative methodologies, and that countries in the North seem better resourced to conduct LA research.

In a context where technologies are omnipresent and mediate human behaviours across all spheres of life, the community of learning analytics in higher education is fast growing and is attracting attention and research efforts worldwide. While this is a positive feature, this paper expresses cautions about the matter. Critical studies of LA are needed so as to interrogate aspects such as data collection and analysis, and implications for students, teachers, managers, researchers and the academic community in general.

Also, concerns and challenges identified in the analysis invite us to revisit questions about the location of *power* in LA; the *marketisation of education* and the exploitation of data for business-like practices; *accountability and audit processes* that promote a conception of educational processes based on metrics; *surveillance* and the promotion of an Orwellian society in which students and teachers feel they are being surveilled; *governance and management of data* at institutional and national levels and their connection with educational policies; the *North-South divide* and the ways in which certain knowledges about technologies, learning and LA are imposed upon countries with fragile economies; and *educational research* and the imperative of strengthening and extending interdisciplinary theories and combined research methods to understand learning in new ways.

While it cannot be denied that technologies have created new environments for learning, through which students approach curriculum content and interact with others in a virtual way, the extent to which the LA data generated, gathered, and analysed actually corresponds to learning remains unclear. Complex learning processes might be underplayed in the data mining analytical techniques associated with LA so that the suggestion that LA has shifted away from a technological focus towards a more educational focus (Ferguson 2012) deserves to be revisited.

In further examining issues on LA, and aligned with previous literature (Daniel 2015; Viberg et al. 2018), it is possible to venture a distinction between a *practice-based community* led by management units within higher education institutions and an *academic community* whose object of research study is LA as such. In other words, while managers and practitioners usually deal with learners’ data in an everyday basis and develop strategies to improve student performance, prevent dropouts and predict completion rates, academics within the field of LA aim critically to examine both the technological tools mediating learning, the mathematical models, and the research methods in use so as to promote and theorise learning. For both communities, LA have become a powerful tool to inform and improve learning through concrete interventions and actions.

Across both communities (practical and academic communities), there is a shortage of papers devoted to developing or expanding educational theories about students’ learning (Ferguson and Clow 2017; Leitner et al. 2017; Viberg et al. 2018). This finding resurrects the issue as to the extent to which LA is about learning as such. Most of the empirical studies on LA seem focused on collecting data, new ways of analysing them, and the development of tools to support students’ learning so exhibiting a rather pragmatic profile. The papers examined here within the thematic analysis were clear that educational and learning theories are insufficiently present in LA research. The role of educational theorists and critical approaches in understanding learning in its complexity are, therefore, crucial in overcoming this pending challenge.

Finally, an undue emphasis on metrics and quantification in research on LA legitimises a technocratic perspective on learning that reinforces audit arrangements and a managerial discourse on learning in higher education. In the process, key learning issues are likely to be underplayed.

## Recommendations and limitations

An active involvement of both teachers and students in both contributing to design the learning environments and in assessing learning seem crucial. In other words, while practitioners, managers and academics are important stakeholders in the LA community, the presence of teachers and students needs to be secured and reinforced (Kollom et al. 2020).

Also, joint and coordinated work between institutional researchers, managers and academics is necessary so as to include a theoretical dimension. This will help in using pedagogy-based approaches and educational theories in understanding learning (rather than a data-driven approach only).

In addition, a full development of LA would lie in a combination of quantitative and qualitative analyses (Al-Mahmood 2020). Qualitative studies could help in overcoming some of the main challenges that LA face such as the simplification of learning processes or the critique that LA is insufficiently sensitive to the time and place of the students’ learning. Also, qualitative techniques might help in examining the teachers, students, managers and authorities’ perceptions of LA, the ways in which students and teachers could be more actively involved, the ways in which privacy and confidentiality can be maintained, and the ways in which data could be better used to promote learning.

 This paper being a literature review, there are limitations to be noted:

- The number of analysed papers is limited since it included only WoScc core collection and SciELO indexes. This decision was based on the fact that both WoScc and SciELO databases share the same Clarivate Analytics’ Web of Science platform so helping in standardising the search. Future research might also consider SCOPUS, other locally recognised indexations, and books.
- The span of time for the search (2013–2019) is also limited. However, given that one of the arguments of this paper is based on a seminal paper by Ferguson published in 2012, it was considered that this span of time is appropriate.

Given these limitations and the analysis performed, the discussion and interpretations contained in the paper cannot be generalised to the whole LA community. A detailed qualitative analysis of different types of publications might help in understanding the extent to which LA investigates learning as such.

## Supplementary Information

**Additional file 1.** Learning Analytics higher education 2013–2019.

## Data Availability

Database has been uploaded as a Additional file 1.

## Footnotes

1 As mentioned later, only the following research categories were included in the search: Education & Educational Research, Education Scientific Disciplines, Psychology Educational and Education Special.

2 The SciELO database does not include index collections.
